# Supplementary material for: Transcriptional Profiling to Assess the Effects of Biological Stimulant Atlanticell Micomix on Tomato Seedlings Under Salt Stress
Source: Plants (Basel). 2025 Apr 11;14(8):1198. doi: 10.3390/plants14081198 (PMC12030531; doi:10.3390/plants14081198)
Supplement: Supplementary file 1 [file plants-14-01198-s001.zip › Supplemental_FiguresFINAL_FINAL.pdf]

Figure S1

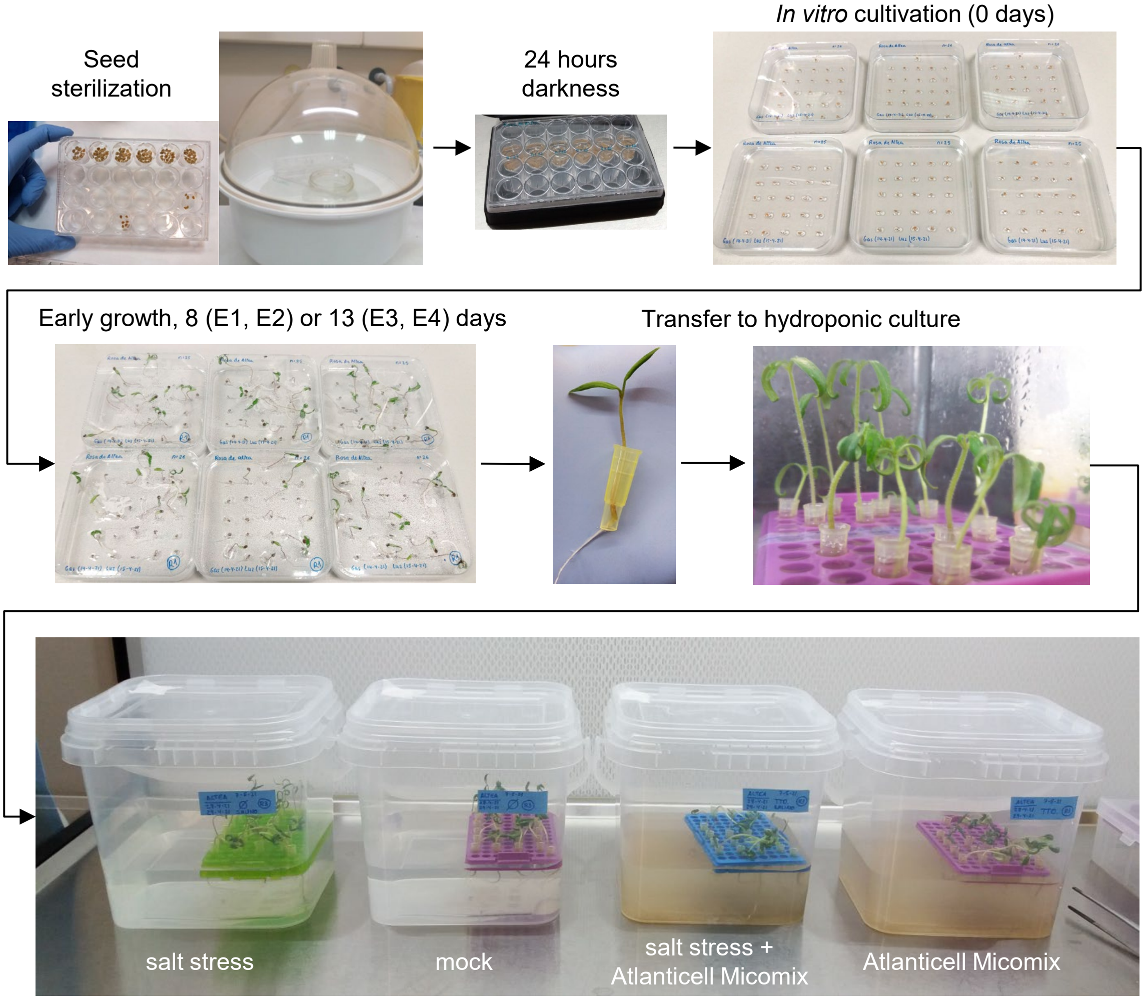

Figure S1. Experimental design used in this work. Four different experiments (E1 to E4) were carried out with four treatments each to evaluate the effect of salt stress and the application of the Atlanticell Micomix (AMM) solution (see Materials and Methods).

Figure S2

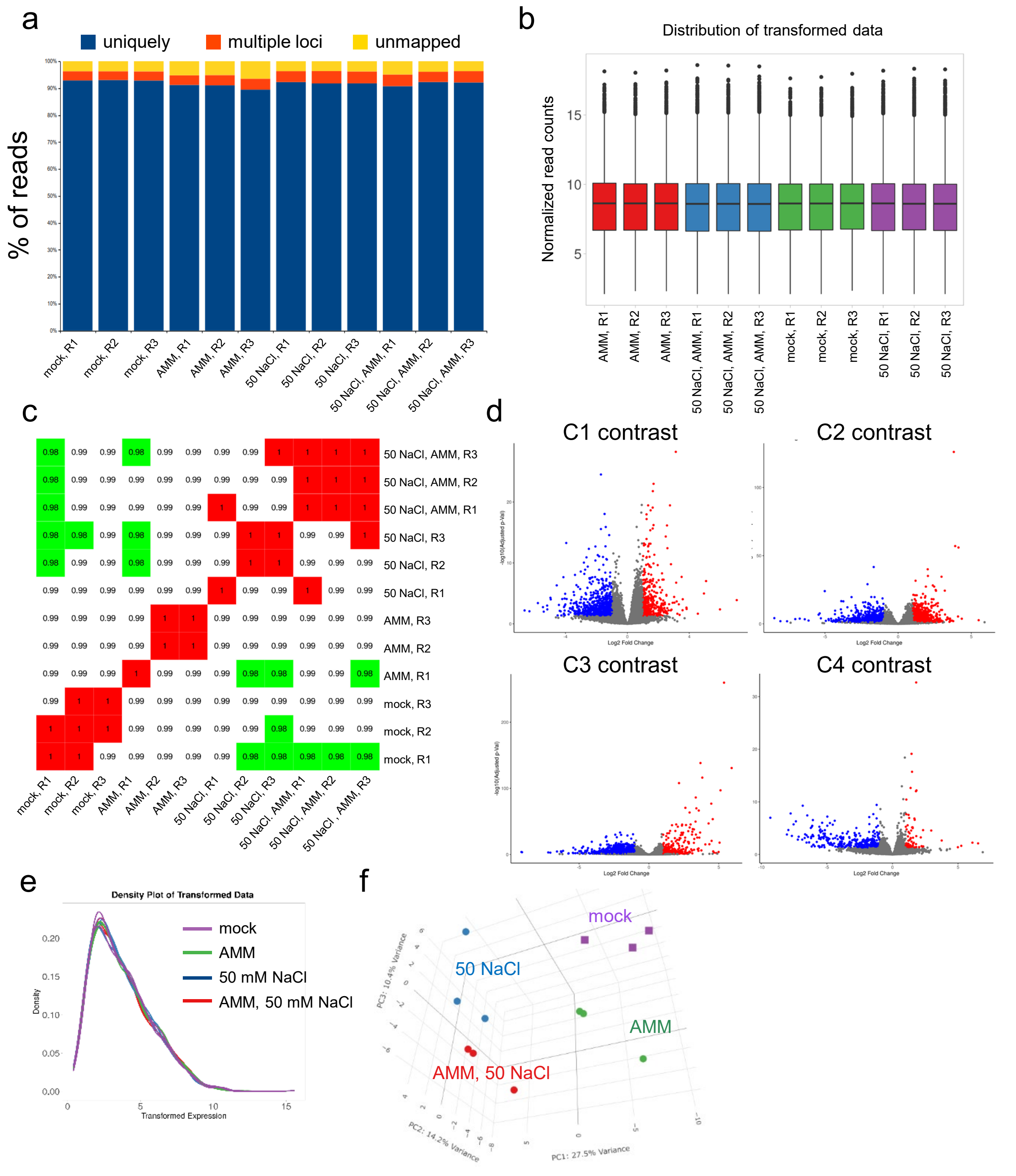

Figure S2. Statistics of RNA-Seq analysis. (a) Distribution of mapping data, percentage of reads that map uniquely, multiple times, and not mapped, to tomato SL4.0 genome. (b) Normalized boxplot of counts per million (CPM). (c) Correlation matrix. (d) Volcano plots of the studied contrasts. The red dots indicate up-regulated differentially expressed genes (DEGs) and the blue dots are used for downregulated DEGs with log2FC > |1| and FDR<0.05. (e) Density plot of ncRNA expression in our dataset. (f) Principal component analysis of the RNA-Seq results from non-coding RNA.

Figure S3

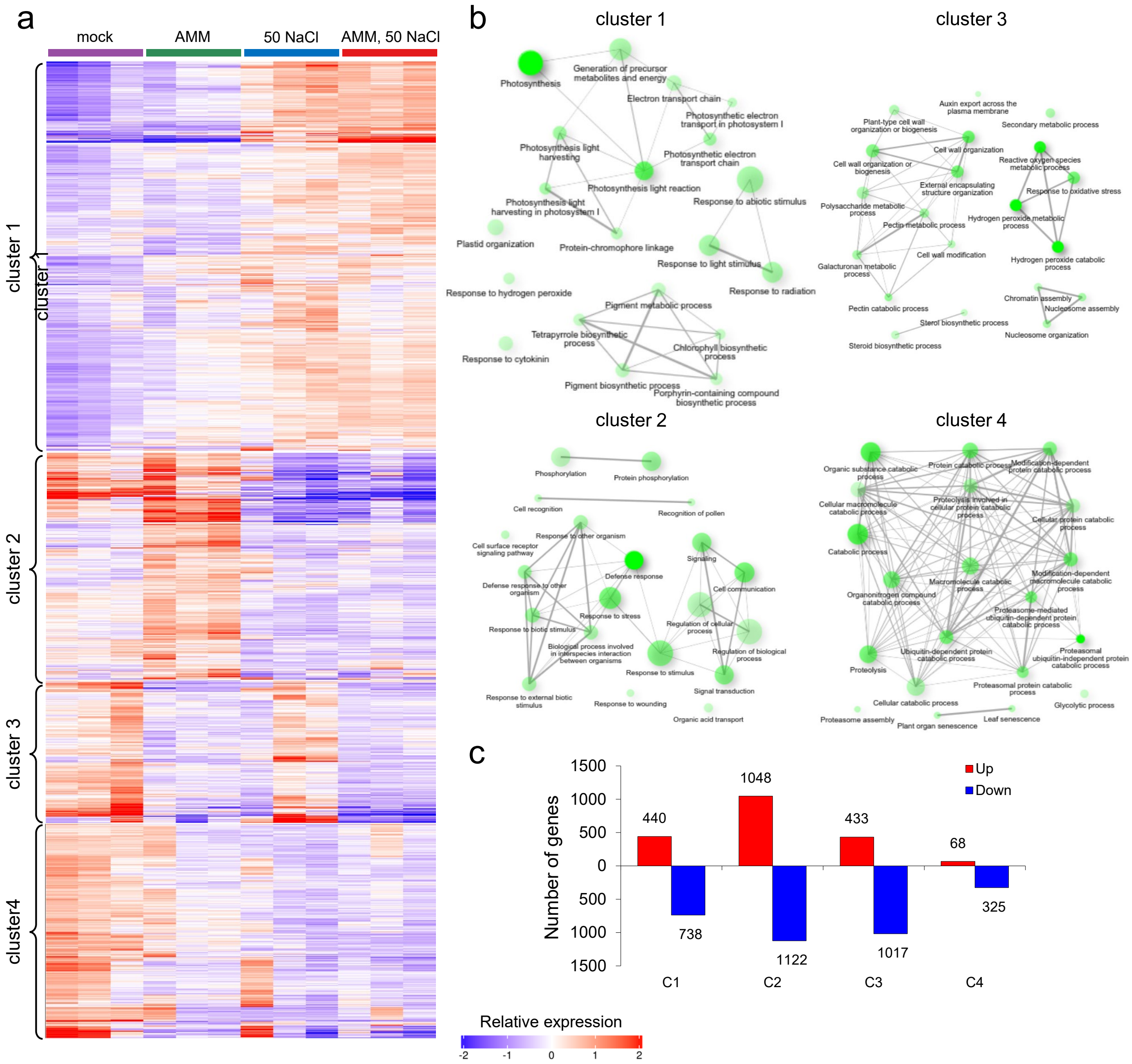

Figure S3. Expression analysis of RNA-Seq data (cont.). (a) K-means clustering of 6,600 most variable genes (SD>0.5). Predicted clusters are grouped according to their expression profile. Expression values are relative and adjusted to −1 (blue) and +1 (red). (b) Enrichment networks of the different GO BP terms of the different clusters found. The GO terms were reduced using the Revigo tool, with default parameters. The intensity of red reflects the FDR enrichment value in the analysis, as well as size of the bubble corresponds to the LogSize value for the GO Term. (c) Number of DEGs obtained in each comparison.

Figure S4

a

b

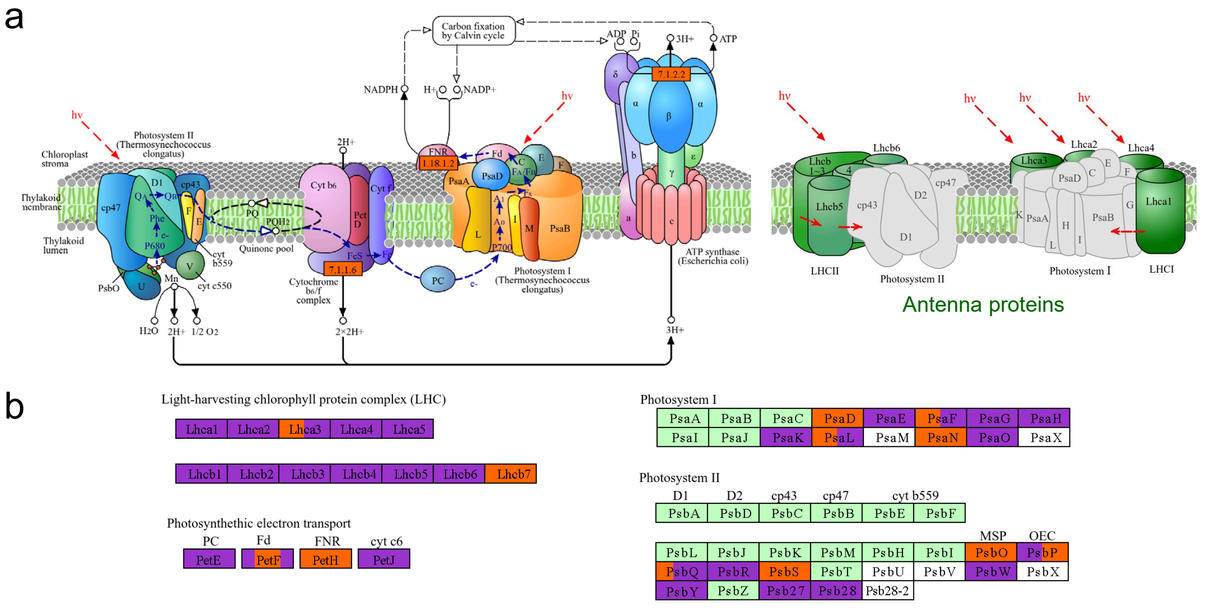

c

Light-harvesting complex II (LHCII)

	mock	AMM	NaCl	AMM NaCl
LHCB1-1	42	58	82	77
LHCB2.1	220	256	456	503
LHCB6B	761	953	1391	1642
LHCB4.2/4.1	2007	2712	4015	4396
LHCB3B	1177	1627	2415	2776
LHCB6A	645	885	1205	1445
LHCB5	2570	3638	4853	5703
LHCB3A	414	619	981	1021
LHCB1B	987	1605	1916	2405
LHCB2.2/2.4/2.1	2224	3379	4341	4659
LHCB1A	654	1171	1455	1598

Photosystem II

	mock	AMM	NaCl	AMM NaCl
PSBQ1/2	769	910	1371	1478
PPL1	61	74	122	141
PSBP	65	84	118	135
PSBRA	1663	2165	3005	3033
PN3	20	26	41	39
PSB28	87	126	160	169
PSBY	1474	2144	2706	2936
PSBW	87	128	208	183
PN1	101	151	181	169
PPD6	47	76	95	100
PSB27-1	64	113	158	163

Light-harvesting complex I (LHCI)

	mock	AMM	NaCl	AMM NaCl
LHCA2	1690	2108	3002	3662
LHCA4A	1005	1318	1989	2327
LHCA3B	150	194	345	359
LHCA1	325	518	862	878
LHCA4B	167	270	466	461
LHCA5	93	136	170	172
LHCA6	48	80	109	112

Photosystem I

	mock	AMM	NaCl	AMM NaCl
PSAK	510	666	1174	1084
PSAFA	206	236	385	412
PSALB	347	431	628	732
PSAH1	186	277	454	514
PSAH2	143	187	315	329
PSAH1/2	825	1042	1489	1525
PSAG	448	599	886	901
PSAO	326	427	610	642
PSAE1	1080	1490	2067	2126
PSAE2	336	480	650	673

Photosynthetic electron transport

	mock	AMM	NaCl	AMM NaCl
PETJ	7	8	12	10
PETE1/DRT112	1064	1410	2009	2218
FD1	5	8	19	26
PNSB4	64	89	136	131
FD2A	1765	2504	3055	3013

Relative expression: min – Max

d

	mock	AMM	NaCl	AMM NaCl
NOT	87	75	28	36
SlNCED2	16	8	5	5
SlCCD8	7	1	1	0

	mock	AMM	NaCl	AMM NaCl
SlTIP2;3	47	16	80	11
SlTIP1;2	10	4	20	4
SlPIP2;4	39	13	23	10

Figure S4. Deregulation of genes which are associated with photosynthesis and drought stress. (a) Photosynthesis pathway as annotated in KEGG. (b) Genes expressed are indicated in orange, and those in purple indicate deregulation. (c) Expression of DEGs associated with photosynthesis. (d) Expression patter of enzymes involved in abscisic acid (ABA) biosynthesis and aquaporin-encoding genes, respectively Grey/orange in c, d indicate normalized CPM values in each row, where orange indicates the highest abundance of transcripts. Gene annotations from are found in Table S4b, c.

Figure S5

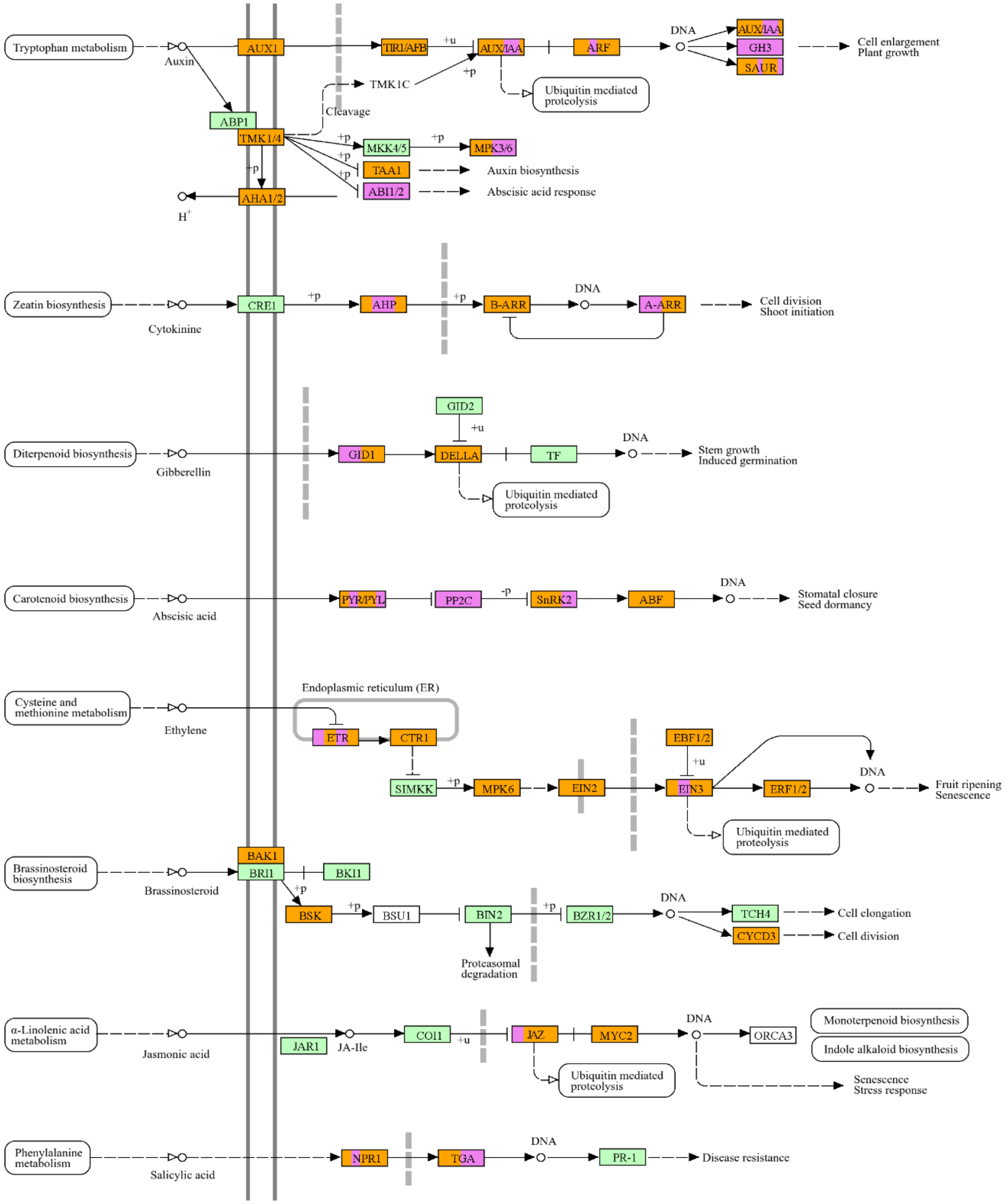

Figure S5. Deregulation of genes which are associated with plant hormone transduction pathways. Genes expressed are indicated in orange, and those in purple indicate deregulation. Figure has been modified from the KEGG database.

Figure S6

Solyc ID	Primer	5'-3' sequence	Amplification (pb)	PCR efficiency
Solyc08g021820	IAA29q-F	TTAATGCCACTTGTCACAAGGA	115	112.3
	IAA29q-R	TCCAACCTGCATGACCTTGA		
Solyc08g069040	LePrx52q-F	CCATGATTGCTTTATTCAGGGT	120	113.7
	LePrx52q-R	ACAGTGTCATATCCATCTCCAGC		
Solyc10g007600	LeGLO2q-F	GGTGCATCAGGCGTGTTTATT	113	118.2
	LeGLO2q-R	AACGACAGCCACTCAATGCC		
Solyc08g008280	WRKY53q- F	CCAAGATCATCATGCGCTCA	211	112.9
	WRKY53q-R	CCAACAGTTTTGCATGTGACG		
Solyc12g005370	LePrx102q-F	TCATGATTGTTTTGTTAGGGGT	202	111.4
	LePrx102q-R	AACGGTGACATCTCTTGCAA		
Solyc03g111010	GADPHq-F	GCTCCAATGTTTGTTGTTGGTG	115	116.8
	GADPHq-R	AATCTGTCATTGATAACCTTGGC		

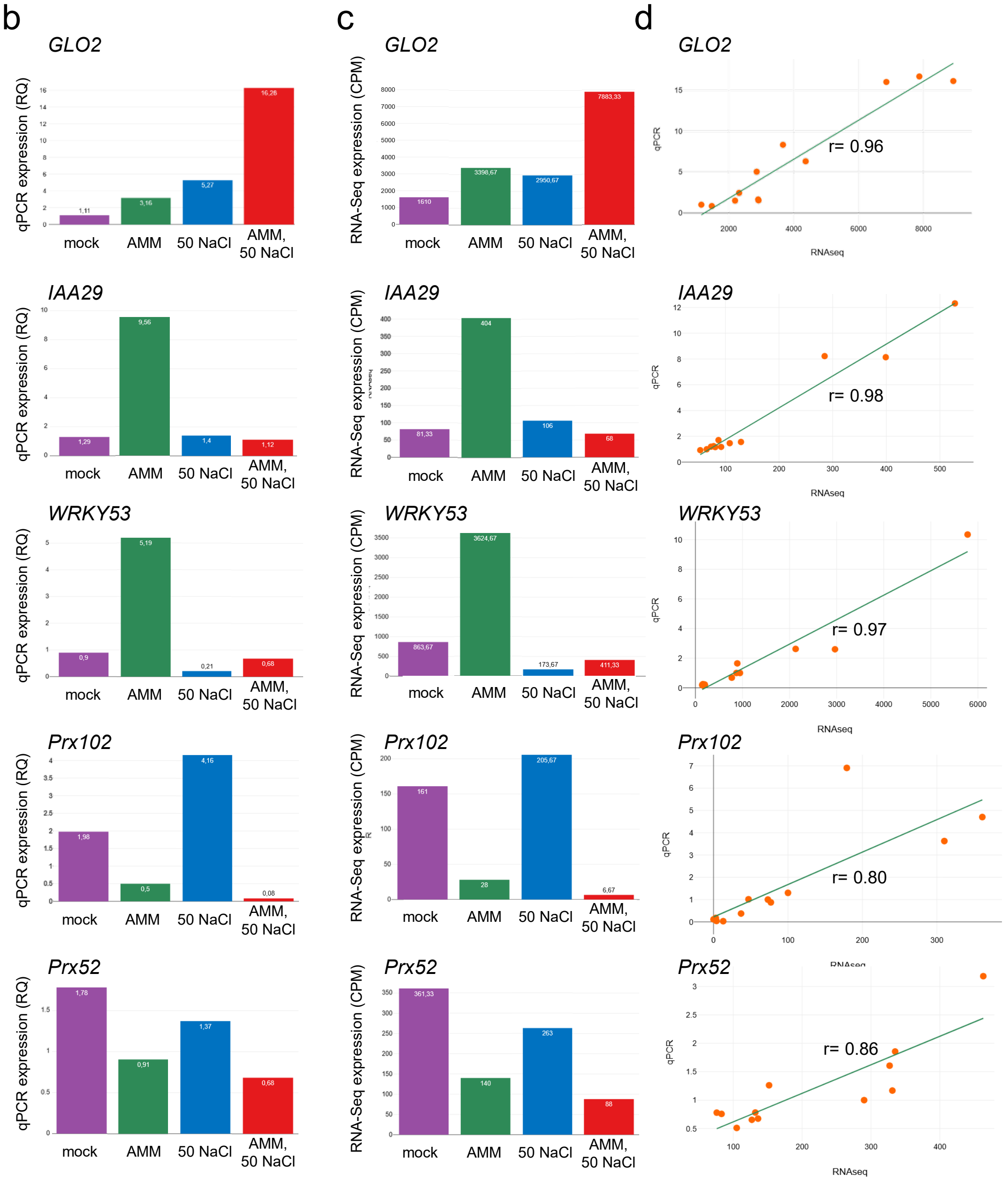

Figure S6. Validation of RNA-Seq results through RT-qPCR. (a) Table of primers used for amplification of selected genes in RT-qPCR. (b) Results of qPCR expression levels (RQ) and (c) results of RNA-Seq expression levels (CPM) in the five selected genes under different conditions. (d) Correlation between qPCR and RNA-Seq results.

Figure S7

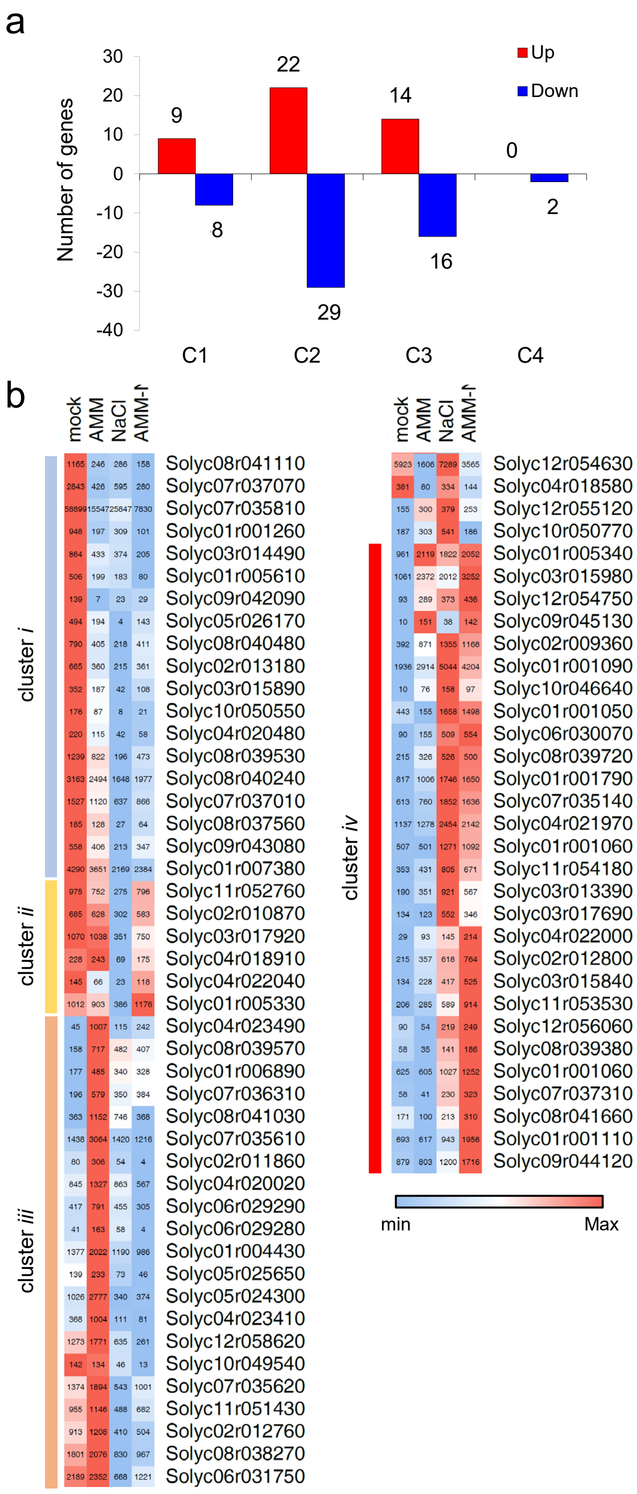

Figure S7. Expression analysis of ncRNAs. (a) Deregulated ncRNAs in the different treatments (log2FC > |1| and FDR<0.05). (b) Clustering analysis of deregulated ncRNAs according to their average expression (in CPM) in the different treatments. Colors indicate normalized CPM values in each row, where blue and red indicate the lowest and highest abundance of transcripts, respectively.
